# 931. Host PFKFB3-dependent glycolytic reprogramming as a broad-spectrum antiviral strategy

**DOI:** 10.1093/ofid/ofad500.976

**Published:** 2023-11-27

**Authors:** Shuofeng Yuan, Zi-Wei Ye, Hin Chu

**Affiliations:** The University of Hong Kong, Hong Kong, Hong Kong; The University of Hong Kong, Hong Kong, Hong Kong; The University of Hong Kong, Hong Kong, Hong Kong

## Abstract

**Background:**

The current therapeutic arsenal against viral infections remains poor, with limited spectrum of coverage and appears inadequate to face the emergence of pandemic and antiviral resistance. This study aims to develop a broad-spectrum antiviral strategy for rapid epidemic control and early treatment with better clinical outcome.

**Methods:**

To investigate the in vivo change of glucose homeostasis under infectious circumstances, we carried out a time-course monitoring of blood glucose concentration in pdmH1N1-infected BALB/c mice and revealed that impairment of glucose tolerance and insulin sensitivity were caused when compared with the mock-infection control. To explore the change of gene expression file upon virus infection, we first plotted the pdmH1N1 virus replication kinetics in human primary bronchial/tracheal epithelial cells (hBTECs), followed by mRNA gene expression profiling of the key glycolytic enzymes at 0hpi, 6hpi and 24hpi, respectively. To discover potential enzymatic inhibitors/modulators of host glycolysis for antiviral treatment, we screened a small molecular compound library with 344 glycolysis compounds on pdmH1N1 infected hTBECs, followed by secondary validation to confirm the dose-dependent virus inhibition at non-toxic concentrations.

**Results:**

Significant higher blood glucose level was detected in infected-mice with either glucose or insulin supplement, which suggested that host glucose metabolism, in particular the glycolysis homeostasis, were disrupted upon influenza virus infection. We also identified Glucose transporter 4 (GLUT4), enolase 2 (ENO2), monocarboxylate transporter 2 (MCT2), MCT4 and 6-phosphofructo-2-kinase (PFK2, also named PFKFB3) the most perturbed genes at 24hpi. KAN0438757, a potent and selective inhibitor of the metabolic kinase PFKFB3, was shown to be antiviral effective against both H1N1 and EV-A71 viruses in human primary cells. Moreover, Kan exhibited broad-spectrum antiviral activity in ZIKV-infected Huh7 cells and SARS-CoV-2 infected Caco-2 cells.

**Figure:**
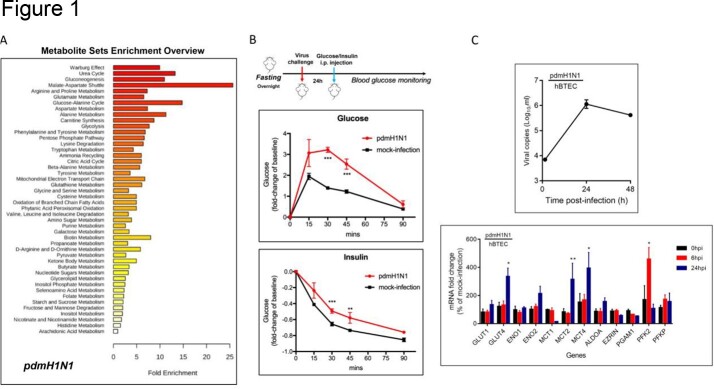

Metabolic profiling reveals significant perturbations of host glucose homeostasis after influenza A virus infection.

**Conclusion:**

Host PFKFB3-dependent glycolysis pathway with broad-relevance to multiple virus replications, which might be a vulnerable host target for antiviral intervention.

**Disclosures:**

**All Authors**: No reported disclosures

